# Postoperative Astigmatism after Keratoplasty: A Systematic Review Meta-Analysis Based on PRISMA

**DOI:** 10.3390/jcm13113306

**Published:** 2024-06-04

**Authors:** Magdalena Kijonka, Anna Nowińska, Edward Wylęgała, Adam Wylęgała, Ewa Wróblewska-Czajka, Katarzyna Kryszan, Bogdan Dugiełło, Bogusława Orzechowska-Wylęgała

**Affiliations:** 1 Chair and Clinical Department of Ophthalmology, School of Medicine in Zabrze, Medical University of Silesia in Katowice, District Railway Hospital, 40-760 Katowice, Poland; anna.nowinska@sum.edu.pl (A.N.); ewylegala@sum.edu.pl (E.W.); adam.wylegala@gmail.com (A.W.); ewaw8@wp.pl (E.W.-C.); kkryszann@gmail.com (K.K.); bogdan.dugiello@gmail.com (B.D.); 2 Department of Ophthalmology, District Railway Hospital in Katowice, 40-760 Katowice, Poland; 3 Health Promotion and Obesity Management, Pathophysiology Department, Medical University of Silesia in Katowice, 40-752 Katowice, Poland; 4 Department of Pediatric Otolaryngology, Head and Neck Surgery, Medical University of Silesia, 40-760 Katowice, Poland; borzechowska-wylegala@sum.edu.pl

**Keywords:** postoperative astigmatism, post-keratoplasty astigmatism, anterior lamellar keratoplasty (ALK), deep anterior lamellar keratoplasty (DALK), posterior lamellar keratoplasty, endothelial keratoplasty (EK), penetrating keratoplasty (PK), Descemet’s stripping endothelial automated keratoplasty (DSEAK), corneal transplant, refractive surgery, laser in situ keratomileusis (LASIK), femtosecond LASIK

## Abstract

**Background**: The number of corneal transplants is rising, with the aim to treat a spectrum of diseases ranging from dystrophies to corneal opacities caused by trauma or chemical burns. Refractive outcomes after this procedure are often impossible to predict and associated with high levels of astigmatism. However, there are many techniques that affect the reduction of astigmatism and improve the quality of life of patients. **Objectives**: The aim was to compare the improvement in postoperative visual acuity (logMAR) and amount of corneal astigmatism (Diopters) after corneal keratoplasty in patients who additionally underwent a surgical procedure, which affects the reduction in postoperative astigmatism, and to determine the most effective method. **Search Methods and Selection Criteria:** A thorough search was carried out across online electronic databases including PubMed, Embrase, Ovid MEDLINE, Scopus, and Web of Science, using combinations containing the following phrases: postoperative astigmatism, post-keratoplasty astigmatism, anterior lamellar keratoplasty (ALK), deep anterior lamellar keratoplasty (DALK), posterior lamellar keratoplasty, endothelial keratoplasty (EK), penetrating keratoplasty (PK), corneal transplant, keratoplasty, refractive surgery, kerato-refractive surgery, laser in situ keratomileusis (LASIK), and femtosecond LASIK. This was to determine all English-language publications that discuss refractive operations for postoperative or post-keratoplasty astigmatism. These bibliographies were searched for English-language publications published between 2010 and 2023. We proceeded to review each detected record’s reference list. **Data Extraction:** Study characteristics such as study design, sample size, participant information, operations performed, and clinical outcomes were all extracted. **Data Statistical Analyses:** The Comprehensive Meta-Analysis software (version 3.3.070, 2014) was used to perform the analysis. The threshold of 0.05 for *p*-values was considered statistically significant. All effect sizes are reported as standardized differences (Std diff) in means with a 95% confidence interval (CI) and visualized graphically as forest plots. Publication bias is presented as a funnel plot of standard error by Std diff in means. Four methods were used to evaluate the heterogeneity among the studies: Q-value, I^2^, chi-square (χ^2^), and tau-squared. **Main Results:** We included 21 studies that randomized 1539 eyes that underwent corneal transplantation surgery either by PKP, DALK, or DSEAK techniques. The results showed the most significant improvement in the visual acuity and significant decrease in the corneal astigmatism after laser surgery procedures, like femtosecond-assisted keratotomy after DALK and PKP and LASIK after DSEAK.

## 1. Introduction

The goal of lamellar keratoplasty (LK) is to restore damaged corneal tissue partially or entirely. The posterior stroma is preserved by anterior lamellar keratoplasty (ALK). ALK has several benefits, such as lowering the possibility of endothelial graft rejection, maintaining structural integrity, and lowering possible intraoperative problems related to open-sky surgeries. Nevertheless, the patient’s quality of vision may be diminished by interface scarring or haze that may heal from the physical dissection of the donor tissue and recipient bed. In recent times, advancements in automation, surgical methods, and equipment have enhanced the effectiveness of ALK surgeries as well as their visual results [1].

In the treatment of corneal ectasia and other disorders of the corneal stroma, deep anterior lamellar keratoplasty (DALK) is becoming more and more preferred over penetrating keratoplasty (PK) [2]. In addition, different techniques are used, including femtosecond laser-assisted anterior lamellar keratoplasty, which is a safe and effective surgical method providing satisfactory graft survival rates [3].

Posterior lamellar keratoplasty, i.e., endothelial keratoplasty (EK), makes a little limbal incision, replacing the damaged corneal endothelium with healthy donor tissue while keeping the patient’s anterior cornea intact. This surgical method has several advantages over penetrating keratoplasty (PK) since it preserves the recipient’s cornea’s structural integrity and damage resistance. Furthermore, because there are no sutures used during the procedure, faster recovery and better visual results are the outcomes. The following are the several EK types that are currently being investigated [4].

Keratoplasty is a safe and efficient way to restore vision. However, there is a considerable chance that patients would experience refractive errors even after clear and effective corneal grafts, which will hinder their ability to receive visual rehabilitation. The most important thing in keratoplasty treatments is to keep the graft healthy, although astigmatism limits the amount of visual rehabilitation that can be achieved with otherwise successful corneal grafts. Postoperative high astigmatism continues to be a major contributor to inadequate vision recovery even with advancements in the DALK technique’s standardization and refinement [5].

Following PK, astigmatism >5 D is reported to occur 10–31% of the time. Previous research indicates that following DALK for keratoconus, spherical ranges from −13.0 to +7.0 D, and astigmatism varies from 0.0 to 10.0 D. Comparing DALK to PK, there is a comparable or even greater incidence of acquiring refractive errors despite the latter’s removal of endothelial graft rejection and reduction of endothelial cell death [6,7].

Also, the management of post-keratoplasty astigmatism takes place when sutures remain at the graft–host junction and after all sutures have been removed. Selective suture manipulation, such as suture adjustments and/or suture removal along the steep meridian of astigmatism, is typically used to treat excessive suture-in post-keratoplasty astigmatism. In certain situations, spectacles can offer good visual acuity; in cases of extreme or irregular astigmatism and anisometropia, on the other hand, contact lenses are a better choice. Toric phakic intraocular lenses have recently been recommended. In 8–20% of cases, surgical intervention is necessary to treat post-keratoplasty astigmatism [8].

Furthermore, additional intervention is required in cases of contact lens failure brought on by corneal irregularity, lens intolerance, dry eye, and issues with manual dexterity [9]. This includes incisional keratotomy, wedge resection, laser refractive surgeries, intracorneal segments, and intraocular lens (IOL) implantation [2,10].

Refractive procedures should be carried out in eyes that have had prior keratoplasty once the corneal shape and refraction have stabilized. A minimum of one month should elapse between refractive surgery and all suture removal, with a three-to-six-month period following suture removal being ideal, according to several studies [11].

In the present study, we review the available surgical methods applied for the management of postoperative astigmatism following anterior and posterior lamellar keratoplasty.

## 2. Methodology

### 2.1. Search Strategy

A thorough search was carried out across online electronic databases including PubMed, Embase, Ovid MEDLINE, Scopus, and Web of Science, using combinations containing the following phrases: postoperative astigmatism, post-keratoplasty astigmatism, anterior lamellar keratoplasty (ALK), deep anterior lamellar keratoplasty (DALK), posterior lamellar keratoplasty, endothelial keratoplasty (EK), penetrating keratoplasty (PK), corneal transplant, keratoplasty, refractive surgery, kerato-refractive surgery, laser in situ keratomileusis (LASIK), and femtosecond LASIK. This was to determine all English-language publications that discuss refractive operations for postoperative or post-keratoplasty astigmatism. These bibliographies were searched for English-language publications published between 2010 and 2023. We proceeded to review each detected record’s reference list.

### 2.2. Clinical Outcomes

In the present study, uncorrected distance visual acuity (UDVA) (logMAR) and topographic astigmatism (D) at the end of follow-up were chosen as the outcome. They are more objective for evaluating postoperative astigmatism compared to manifest refraction. 

### 2.3. Data Extraction

Study characteristics such as study design, sample size, participant information, operations performed, and clinical outcomes were all extracted. 

### 2.4. Data Statistical Analyses

The Comprehensive Meta-Analysis software (version 3.3.070, 2014) was used to perform the analysis. The threshold of 0.05 for *p*-values was considered statistically significant. All size effects are reported as mean and its 95% confidence interval (CI), and values are shown as means (standard deviation, range). Furthermore, four methods were used to evaluate the heterogeneity among the studies: I^2^, chi-square (χ^2^), and forest plot overlap. It was determined which variables are responsible for the distinct heterogeneity by performing subgroup analysis. *p* < 0.05 was established as the significant threshold.

## 3. Results

**Figure 1:**
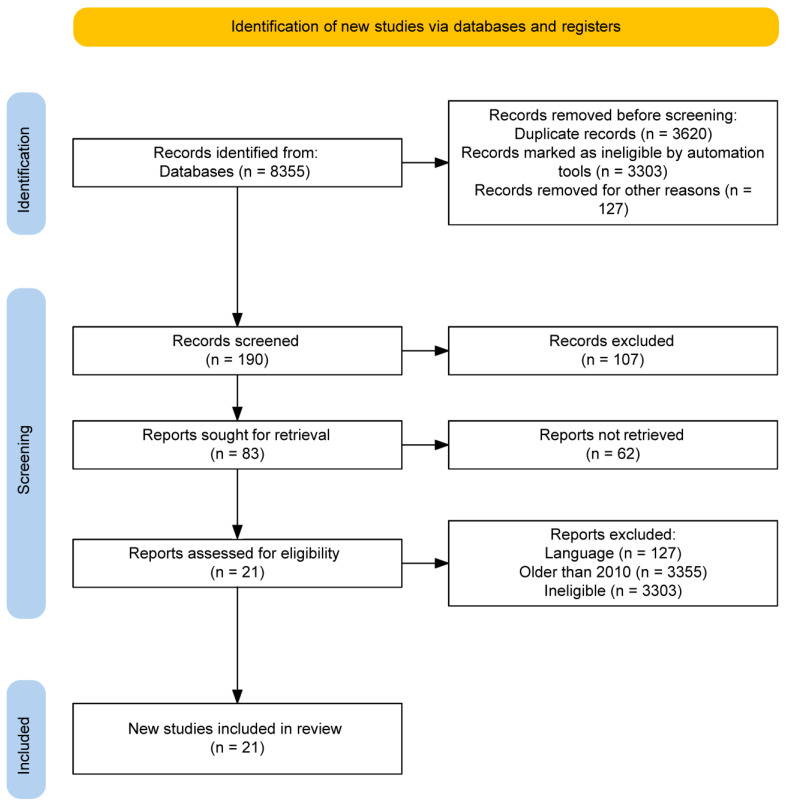
Flow diagram of the publication search process.

There were 8355 potential article citations in the preliminary literature review (Figure 1). Of these citations, 3620 studies were immediately excluded because they were duplicated. Another 3303 studies were excluded, as they were ineligible. Then, 127 studies were excluded, as they were published in languages other than English, according to the exclusion criteria. The full text of the remaining 190 articles was downloaded for a more thorough examination. Following the full-text reading, 107 articles were eliminated. Eventually, 21 previously published papers were chosen based on the inclusion and exclusion criteria of the present study. The inclusion criteria were research dated 2010 or newer, in the English language, and containing eligible research for our main topic. The exclusion criteria were research older than 2010, not in the English language, and containing ineligible research not focused on our main topic. The included 21 studies included 1539 randomized eyes. All studies compared the change in astigmatism and visual acuity after corneal transplantation surgery (PKP or DALK).

As shown in Table 1, raw data were extracted from 21 studies (12 retrospective, 1 retrospective quasi-experimental study, 7 prospective, and 1 interventional case series) that were selected from 190 articles.

In Table 2 we showed the ratio of methods used for refraction correction which were extracted from 21 studies.

### 3.1. The Change in Astigmatism

**Figure 2:**
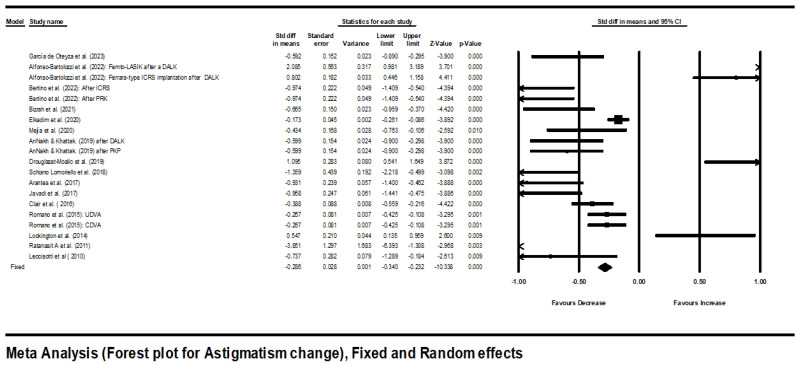
Forest plot for astigmatism change using fixed- and random-effects model [5,12,14,15,16,17,18,19,20,21,22,23,24,26,27,29,30].

As shown in Figure 2, there was an overall statistically significant improvement in astigmatism (diopters). The point estimate with its 95% CI using random effect model was −0.357 (−0.550 to −0.163).

As the mean effect size was heterogeneous (significant Q statistic: Q = 170.4, *p* = 0.001), a random-effects model was used for analysis.

### 3.2. The Change in Visual Acuity

**Figure 3:**
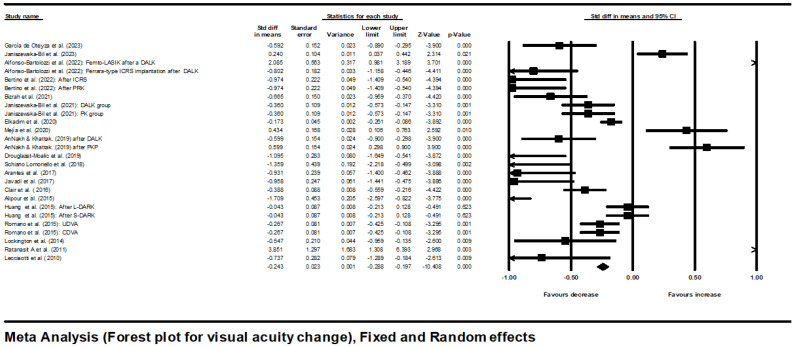
Forest plot for visual acuity change using fixed- and random-effects model [5,6,12,13,14,15,16,17,18,19,20,21,22,23,24,25,26,27,29,30,31].

As shown in Figure 3, there was an overall statistically significant improvement in Visual acuity (logMar). The point estimate with its 95% CI using random effect model was −0.383 (−0.535 to −0.231).

As the mean effect size was heterogeneous (significant Q statistic: Q = 210.9, *p* = 0.001), a random-effects model was used for analysis.

**Figure 4:**
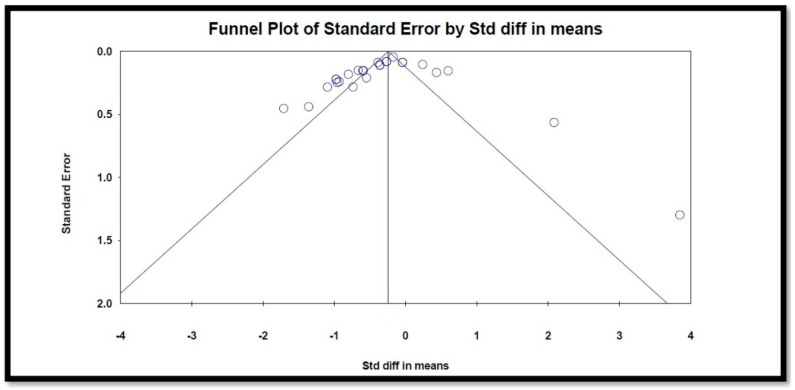
Funnel plot for analysis of publication bias.

A Begg’s funnel plot was used to visually detect the presence of publication bias. The plot displays the results of the studies (*x*-axis) and precision (*y*-axis). The results are the standardized mean differences (effect size), and the precision is the standard error of the standardized mean differences. Each dot of the plot represents a separate study. The middle solid line indicates the overall effect of the meta-analysis. Begg’s funnel plot showed asymmetry (Figure 4), suggesting potential publication bias.

## 4. Discussion

The purpose of this review and meta-analysis was to assess the management of postoperative astigmatism following corneal keratoplasty. We included 21 studies in this review that had 1539 eyes managed for astigmatism after anterior and posterior lamellar keratoplasty and penetrating keratoplasty.

The visual outcomes are comparable between DALK and PK. DALK is a procedure that is mainly performed on young patients suffering from keratoconus who expect excellent visual acuity. Refraction stabilizes up to 6 months after the sutures are completely removed. After this time, vision-correction methods can be considered [32]. Diverse methods of treating astigmatism after PKP or DALK surgery have been described. Glasses as well as hard and soft contact lenses tend to be the primary choices. Nevertheless, visual acuity often remains unsatisfactory [33]. Other approaches mentioned in the present study include surgical procedures such as suture tightening or selective suture removal as well as novel blunt dissection technique [5], intracorneal ring segments (ICRS) including Ferrara-type ICRS implantation [14,22], femtosecond laser-assisted ICRS implantation [13,14,19,24,34], relaxing incisions [23], corneal wedge resection [12,18], photorefractive keratectomy (PRK) [17], and deep intrastromal arcuate keratotomy with in situ keratomileusis (DIAKIK) [20].

Another technique used after PKP and DALK involves astigmatic keratotomy (AK) [26,28], toric intraocular lenses (IOLs) [21,27,30], and miniscleral lenses [25].

According to the majority of current studies, femtosecond-assisted keratotomy appears to be a safe and effective method for significantly reducing corneal astigmatism. Also, there is substantial literature evidence that femto–LASIK is the most successful indication for management of postoperative astigmatism after corneal keratoplasty with excellent outcomes [13,14,16]. In the present studies, the UDVA improved significantly in all eyes at 6 months of femto-LASIK. The visual result is excellent, even though there may be instances of overcorrection and undercorrection. Additionally, ref. [19] reported that femtosecond AK has a similar safety profile for the treatment of suture-out post-DALK and -PKP astigmatism. However, it is more effective for post-DALK astigmatism.

Most of the patients with astigmatism higher than 6 D had residual cylinder less or equal to 3 D, which can be treated by laser excimer ablation or secondary IOL implantation [21]. On a lesser scale, [14] evaluated the outcomes of the femto–LASIK technique in patients who had undergone DALK previously and still had residual astigmatism. After examining ten eyes and following-up after thirty to sixty months, they found no intraoperative or postoperative problems. These findings led to the conclusion that femto–LASIK is a safe and effective treatment for individuals with residual astigmatism following DALK.

The results of the meta-analysis also revealed that the astigmatism was decreased, and visual acuity was increased after the management of astigmatism regardless of the used method. This is in agreement with [35,36]. Additionally, as shown in the results of the present meta-analysis, DALK achieved a better visual acuity compared to PK by calculating logMAR and the BCVA. However, comparable visual outcomes were obtained between PKP and DALK groups at different time points. In agreement with these results, it was found that the BCVA of the DALK group was slightly but non-significantly better than that of the PK group [37]. It was also reported that BCVA showed better visual results compared to those obtained with PK [38]. In addition, it was found that, in contrast to PKP, there were fewer complications in the DALK group, suggesting that DALK is less effective but safer than PKP [35]. Some investigations have shown that PKP achieved better visual acuity [39].

In contrary to high astigmatic error after anterior lamellar keratoplasty, the literature confirms that endothelial keratoplasty procedures (DSEK/DSAEK) induced corneal astigmatism less than 1.00, occurring between 6 and 12 months. This low astigmatism is related to the absence of corneal incisions or sutures that change corneal strength and refraction. DMEK may be the first technique in corneal transplantation that (on average) is considered to be refractive-neutral. The number of articles regarding the correction of refractive error after DSEAK or DSEAK is very limited. The residual refractive error could be corrected with spectacles. This procedure is often performed in elderly patients, so this kind of correction is the most effective for them. The spectacles can be prescribed about 3 months after the procedure because of stabilization of refractive. Other techniques mentioned in the present study include surgical procedures such as photorefractive keratectomy or laser-assisted in situ keratomileusis (LASIK) [29]. Both procedures are effective in correcting residual refractive error after DSAEK, but the control group was small, and the postoperative period was considerably too short (21 months) to observe any possible complications. It requires more research.

### 4.1. Limitations of the Study

1. The main limitation of this review is the lack of standardization due to several different studies having different methods for measuring astigmatism, with different methods such as keratometry, pentacam, and autorefractors included within the present research;
2. There was risk of bias in studies where most research only disseminates good results and successful operations and does not record cases where a corneal transplant failed, or the patch was rejected;
3. A low number of studies were available on the topic;
4. There is a lack of information about the corneal graft in the preoperative and postoperative periods. Graft health, endothelial count, and clear zone all affect the outcome and should be well documented to further explain the results;
5. There was a lack of standardization of research and evaluation of results.

### 4.2. Power Analysis

Power analysis was run by using an Excel calculator created by Tiebel J. (http://osf.io/5c7uz, accessed on 20 May 2024) [40]. Regarding astigmatism, for high heterogeneity, the power (1-β) was 96.8% for the fixed-effects model and 99.7% for the random-effects model. Regarding visual acuity, for high heterogeneity, the power (1-β) was 96.9% for the fixed-effects model and 100% for the random-effects model.

## 5. Conclusions

There are several methods in the literature for repairing refractive problems following corneal keratoplasty. For the elimination of significant corneal astigmatism, laser surgery procedures like femtosecond-assisted keratotomy in DALK and PKP and also LASIK after DSEAK appear to be a safe and effective method. More extensive, long-term research with a higher sample size is needed to accurately evaluate the durability of this surgery and validate its safety.

## Figures and Tables

**Table 1:** Comparative analysis of studies conducted by different authors, examining the outcomes of various surgical methods used to improve visual acuity and refractive astigmatism in patients following penetrating keratoplasty and anterior and posterior lamellar keratoplasty.

Author	No. of Eyes	Study	Method	Best Corrected Visual Acuity (BCVA) in logMAR		Topographical Corneal Astigmatism in Diopters	
				Preoperatively	Postoperatively	Preoperatively	Postoperatively
1. García de Oteyza et al. (2022) [12]	51 eyes	Retrospective quasi-experimental, before and after study	Manual wedge resection was performed after PK	1.25 (0.27) logMAR	0.84(0.50) logMAR	2.68 (4.21) D	0.86 (3.13) D
2. Janiszewska-Bil et al. (2023) [13]	96 eyes	Prospective study	Femto-LASIK after DALK	0.1 ± 0.05 logMAR	0.7 ± 0.05 (12 months) logMAR		
3. Alfonso-Bartolozzi et al. (2022) [14]	10 eyes	Retrospective case series	Femto-LASIK after a DALK	0.13 ± 0.05 logMAR	0.47 ± 0.15 logMAR	−3.88 ± 1.00 D	−0.93 ± 0.39 D
4. Alfonso-Bartolozzi et al. (2023)-Bartolozzi [15]	40 eyes	Retrospective study	Ferrara-type ICRS implantation after DALK	1.39 ± 0.55 logMAR	0.71 ± 0.37 at 6 months logMAR	−6.86 ± 2.62 D	−2.33 ± 1.09 D at 6 months
5. Bertino et al. (2022) [16]	30 eyes	Prospective interventional study	Femtosecond laser-assisted ICRS implantation combined with PRK post penetrating keratoplasty	1.16 ± 0.37 logMAR	0.69 ± 0.40 logMAR after ICRS 0.34 ± 0.29 12 months after PRK	7.10 ± 1.13 D	4.61 ± 1.61 D after ICRS 2.58 ± 1.49 D after PRK
6. Bizrah et al. (2021) [17]	54 eyes	Retrospective interventional case series	Topography-guided photorefractive keratectomy (TG-PRK) after PK	0.96 ± 0.06 logMAR	0.46 ± 0.05 logMAR	5.24 ± 0.36 D	2.98 ± 0.34 D at the final follow-up
7. Sorkin et al. (2021) [8]	150 eyes	Retrospective, comparative, pairwise-matched case series	Femtosecond astigmatic keratotomy (FSAK) (*n* = 75) Manual astigmatic keratotomy (AK) (*n* = 75) following PK or DALK	Manual AK: 1.09 ± 0.47 logMAR FSAK: 1.16 ± 0.45 logMAR	Manual AK: 1.06 ± 0.47 logMAR FSAK: 0.89 ± 0.51 logMAR	Manual AK: 8.70 ± 3.30 D FSAK: 9.40 ± 2.80 D	Manual AK: 6.20 ± 3.90 D FSAK: 4.80 ± 3.20 D FSAK had superior visual and keratometric outcomes compared with manual AK
8. Elkadim et al. (2020) [5]	40 eyes	Retrospective interventional case series	Novel blunt dissection technique after modified DALK	0.21 ± 0.20 logMAR	0.11 ± 0.13 logMAR (*p* < 0.001)	6.32 ± 2.56 D	2.61 ± 1.05 D (*p* < 0.001)
9. Mejía et al. (2020) [18]	39 eyes	Retrospective observational study	Corneal wedge resection after DALK	0.35 ± 0.01 logMAR	0.57 ± 0.02 at 12 months logMAR	7.99 ± 0.25 D	2.5 ± 0.3 D at 12 months and remained stable thereafter (a mean follow-up of 76.3 months)
10. AnNakh and Khattak. (2019) [19]	50 eyes	Retrospective, comparative, interventional study	Femtosecond laser-assisted astigmatic keratotomy for suture-out post DALK or PKP corneal astigmatism	0.57 ± 0.48 logMAR	0.38 ± 0.29 logMAR	4.55 ± 2.52 D	3.17 ± 1.68 D
11. Drouglazet-Moalic et al. (2019) [20]	20 eyes	Prospective study	Deep intrastromal arcuate keratotomy with in situ keratomileusis (DIAKIK) after keratoplasty.	1.12 ± 0.42 logMAR	0.58 ± 0.23 logMAR	−5.01 ± 4.35 D	−1.54 ± 2.42 D
12. Schiano Lomoriello et al. (2018) [21]	10 eyes	Prospective, noncomparative, interventional case series	A customized toric IOL implantation after DALK and cataract surgery	0.55 ± 0.29 logMAR	0.14 ± 0.12 logMAR	4.92 ± 1.99 D	3.80 ± 1.60 D
13. Arantes et al. (2017) [22]	25 eyes	Retrospective, longitudinal study	Intrastromal corneal ring segments for astigmatism correction after DALK	0.33 (±0.10) logMAR	0.20 (±0.09) logMAR	3.87 D	1.90 D
14. Javadi et al. (2017) [23]	24 eyes	Interventional case series	Relaxing incision procedure after DALK	0.26 ± 0.14 logMAR	0.22 ± 0.09 logMAR	6.28 ± 1.20 (range, 4.0 to 9.0) D	3.45 ± 1.80 (range, 0 to 8.50) D
15. St. Clair et al. (2016) [24]	140 eyes	Retrospective interventional case series	Femtosecond laser AK procedures were performed after penetrating keratoplasty (PKP) (*n* = 129) Femtosecond laser AK procedures were performed after deep anterior lamellar keratoplasty (DALK) (*n* =11)	0.47 logMAR ± 0.38 (SD)	0.35 ± 0.31 logMAR	6.77 ± 2.80 D	2.85 ± 2.57 D
16. Alipour et al. (2015) [25]	12 eyes	Prospective interventional case series	Fitting with miniscleral lenses after DALK	1.05 logMar (SD: 0.54)	0.17 logMar (SD: 0.19) with the miniscleral lens.		
17. Romano et al. (2015) [26]	158 eyes	Retrospective noncomparative interventional study	Astigmatic keratotomy or laser refractive surgery after DALK.	UDVA was 20/400 (1.5 ± 0.4 logMAR), CDVA 20/50 (0.7 ± 0.2 logMAR)	UDVA was 20/50 (0.5 ± 0.3 logMAR), CDVA to 20/25 (0.09 ± 0.1 logMAR)	4.7 ± 2.6 D	2.9 ± 1.3 D
18. Lockington et al. (2014) [27]	26 eyes	Retrospective case series	Cataract surgery and toric IOL (Acrysof SN60AT or T-flex 623T/573T) implantation post keratoplasty	1.12 ± 0.67 logMAR	0.45 ± 0.39 logMAR	−5.49 ± 3.72 D	−2.61 ± 2.10 D
19. Kubaloglu et al. (2011) [28]	44 eyes	Prospective, comparative, interventional case series	Standard manual 1-pair, 90-degree, and 90% corneal thickness AK incisions after DALK or PK 44 eyes (20 eyes underwent DALK; 24 eyes underwent PK)	0.88 ± 0.20 logMAR (in DALK) 1.0 ± 0.34 logMAR (in PK)	0.54 ± 0.26 logMAR (in DALK) 0.53 ± 0.26 logMAR (in PK)	6.24 ± 0.75 D (in DALK) 6.48 ± 1.45 D (in PK)	After 6 months follow-up 3.53 ± 1.62 (in DALK)D 3.31 ± 2.17 (in PK)D
20. Ratanasit A et al. (2011) [29]	5 eyes	Retrospective case series	Photorefractive keratectomy (PRK) or laser-assisted in situ keratomileusis (LASIK) with intraoperative mitomycin C after DSAEK	0.68 ± 0.15 logMAR	0.78 ± 0.13 logMAR	7.05 ± 2.03 D (range, 4–9.75 D)	1.08 ± 0.58 D (range, 0–2.50 D)
21. Leccisotti et al. (2010) [30]	16 eyes	Retrospective, consecutive, noncomparative, single-surgeon series	Phacoemulsification and IOL implantation after DALK	0.48 logMAR	0.13 logMAR	10.32 D	2.57 D

**Table 2:** The ratio of methods used for refraction correction.

	Methods Used for Refraction Correction	*n*	%
1.	Manual wedge resection was performed after PK	51	4.904
2.	Femto–LASIK after DALK	106	10.192
3.	Ferrara-type ICRS implantation after DALK	40	3.846
4.	Femtosecond laser-assisted ICRS implantation combined with PRK post penetrating keratoplasty	30	2.885
5.	Topography-guided photorefractive keratectomy (TG-PRK) after PK	54	5.192
6.	Femtosecond or manual astigmatic keratotomy following PK or DALK	150	14.423
7.	Novel blunt dissection after modified DALK	40	3.846
8.	Corneal wedge resection after DALK	39	3.750
9.	Femtosecond laser-assisted astigmatic keratotomy after DALK and PKP	50	4.808
10.	Deep intrastromal arcuate keratotomy with in situ keratomileusis (DIAKIK) after keratoplasty	20	1.923
11.	A customized toric IOL implantation after DALK and cataract surgery	10	0.962
12.	Intrastromal corneal ring segments for astigmatism correction after DALK	25	4.404
13.	Relaxing incision procedure after DALK	24	2.308
14.	Femtosecond laser AK procedures were performed after penetrating keratoplasty (PKP)	129	12.404
15.	Femtosecond laser AK procedures were performed after deep anterior lamellar keratoplasty (DALK)	11	1.058
16.	Fitting with miniscleral lenses after DALK	12	1.154
17.	Astigmatic keratotomy or laser refractive surgery after DALK	158	15.192
18.	Cataract surgery and toric IOL (Acrysof SN60AT or T-flex 623T/573T) implantation post keratoplasty	26	2.500
19.	Astigmatic keratotomy after DALK and PK	44	4.231
20.	Photorefractive keratectomy (PRK) with intraoperative mitomycin C after DSAEK	5	0.481
21.	Phacoemulsification and IOL implantation after DALK	16	11.538
	Total	1040	100

## Data Availability

The data used in the review are available upon request.
